# Living arrangements and place of death of older people with cancer in England and Wales: a record linkage study

**DOI:** 10.1038/sj.bjc.6602038

**Published:** 2004-07-13

**Authors:** E Grundy, D Mayer, H Young, A Sloggett

**Affiliations:** 1 Centre for Population Studies, Department of Epidemiology and Population Health, London School of Hygiene & Tropical Medicine, 49-51 Bedford Square, London WC1B 3DP, UK

**Keywords:** palliative care, cancer, place of death, older people, longitudinal, household and family

## Abstract

The main objectives of the study were to (1) see whether the household circumstances of people aged 50 years and over with cancer, and trends in these, differ from those of the rest of the population and (2) whether living arrangements and presence and health status of a primary coresident are associated with place of death among older people dying of cancer and those dying from other causes. The design included prospective record linkage study of people aged 50 years and over included in a 1% sample of the population of England and Wales (the Office for National Statistics Longitudinal Study). The main outcome measures comprised family and household type, and death at home. The household circumstances of older people with cancer were very similar to those of the rest of the population of the same age and both showed a large increase in living alone, and decrease in living with relatives, between 1981 and 1991. The primary coresident of cancer sufferers who did not live alone was in most cases a spouse, with much smaller proportions living with a child, sibling or other person. In all, 30% of spouse, and 23% of other, primary coresidents had a limiting long-term illness. Compared with people who lived alone in 1991, odds of a home death among those dying of cancer between 1991 and 1995 were highest for those who lived with a spouse who had no limiting long-term illness (odds ratio (OR) 2.52, 95% confidence interval (CI) 2.15–2.97) and raised for those living with a spouse with a long-term illness (OR 2.14, CI 1.79–2.56) and those living with someone else who was free of long-term illness (OR 2.13, CI 1.69–2.68). Higher socioeconomic status, both individual and area, was positively associated with increased chance of a home death, while older age reduced the chance of dying at home. The changing living arrangements of older people have important implications for planning and provision of care and treatment for cancer sufferers.

Cancer is a predominant cause of death and in 2001 accounted for 26% of all deaths in England and Wales, compared with 20% attributable to ischaemic heart disease (Office for National Statistics, 2002). In older age groups, even higher proportions of deaths are due to cancer. Improvements in cancer survival, increases in the incidence of some cancers and the ageing of the population also mean that the prevalence of cancer in the population has increased, so there are both more people living with cancer and more people dying from cancer than in the past. In this context, the support of older people with cancer including support to die at home if they wish for those with terminal conditions, is becoming an increasingly important issue for health providers and policy makers. It has recently been suggested, for example, that if more cancer patients were enabled to die at home, the UK National Health Service could make savings of £100 m every year (Burke, 2004). However, information on relevant parameters, such as with whom people with cancer live, is very limited. Here, we investigate the living arrangements of older people, and characteristics of those they live with, and see whether these are associated with chance of a home death.

Coresidents are potential providers of emotional support, domestic help and personal and nursing care for people with cancer, although ability and willingness to provide these supports may depend on characteristics such as health status, age and relationship to the cancer patient. Support from a coresident may influence both the quality of life of cancer sufferers and their need for external services, including in-patient admission. Studies of particular patient groups from a range of settings have reported that cancer patients who live alone report more distress, poorer adjustment to diagnosis and have a poorer quality of life than those living with others (Forsberg and Cedermark, 1996; Akechi *et al*, 1998; Rustoen *et al*, 1999; Sollner *et al*, 1999; Sarna *et al*, 2002). Living arrangements may also influence chance of a home death, for which many people with cancer report a preference (Davison *et al*, 2001; Serra-Prat, Gallo and Picaza, 2001). Local studies of particular patient groups in the UK suggest that people dying from cancer are more likely to be admitted for terminal care if they live alone (Hinton, 1994); similar studies from Belfast, Italy and North America have reported that cancer patients living with a spouse more often die at home (Costantini *et al*, 1993; McWhinney, Bass and Orr, 1995; Davison *et al*, 2001; Gallo, Baker and Bradley, 2001). Several studies have also reported an association between socioeconomic disadvantage and a reduced chance of a home death, although some of these have only used ecological indicators of socioeconomic status (Grande, Addington-Hall and Todd, 1998; Higginson *et al*, 1999).

In Britain and other industrialised countries, recent decades have seen marked changes in the living arrangements of older people, including large increases in the extent of solitary living and declines in the proportion of people living with relatives other than, or in addition to, a partner and never married children. These changes have been greatest in very old age groups; between 1971 and 1991, for example, the proportion of women aged 85 years and over living in households including two or three generations fell from 42 to 21%, whereas the proportion living alone increased from 30 to 49% (Grundy, 1999). It is not known whether trends in the living arrangements of people with cancer have changed to the same extent or whether they differ from those of the rest of the population of the same age. This might be the case if, for example, older people responded to a cancer diagnosis by moving in with relatives, or having a relative move in with them, or if differentials in survival by household circumstances influenced the distribution of the population with cancer by living arrangement.

In this paper, we use data from a large, nationally representative record linkage study, the Office for National Statistics Longitudinal Study (ONS LS), to examine the living arrangements of people with cancer, trends in these living arrangements, characteristics of coresidents of cancer sufferers and associations between living arrangements and death at home.

## STUDY SAMPLE AND METHODS

The ONS LS includes information on census characteristics of sample members, and those they live with, and cancer and death registration data for approximately 1% of the population of England and Wales. The sample size in the ONS LS has remained constant at approximately 540 000 individuals since 1971 (Blackwell *et al*, 2003). The sample was initially drawn from people enumerated in the 1971 Census and has been maintained through the addition of 1% of immigrants and new births. The data may be used both to look at trends over time (comparison of cross-sectional data from successive censuses or deaths in successive periods) and in longitudinal analyses of changes in individual circumstances or length of survival. Linkage of cancer registrations and deaths is achieved via the National Health Service Central Register. Particularly in the age groups considered here, estimated linkage rates are very high (close to 100%) and loss to follow-up low (Hattersley and Creeser, 1995).

We first compare the family and household type of people aged 50 years and over with and without a registered cancer in 1981 and 1991 to see whether the distribution by living arrangements, and trend in living arrangements, was similar for people with cancer and the rest of the population. Sample members included in this analysis were those aged 50 years and over at the relevant census who had been members of the LS for the preceding decade. People with cancer were defined as those with a malignant cancer registered in the previous 10 years (1971–1981 or 1981–1991). Cases with benign growths, *in situ* carcinomas and growths with uncertain, unknown or wrongly coded behaviour (which occurs when a site code is benign but behaviour is coded as malignant) were excluded; these exclusions accounted for under 10% of cases. In the fewer than 5% of cases where more than one cancer registration was recorded, first occurring registration was selected.

The classification of household type was derived from census information on families and households and has been used in other analyses of living arrangements of older people (Grundy, 1999). We classified people as solitary; living just with a spouse (couple only); with a spouse and others (generally children); those with no partner living with a never-married child (lone parent); those in households we term ‘complex’ (people living with relatives other than a spouse or never-married child, such as a married child or a sibling, and those in households containing more than one family, for example, a married couple, daughter and daughter's child); and those in communal establishments such as hospitals or nursing homes. Analyses are restricted to those permanently resident at the place of enumeration (98% of the ONS LS sample).

Secondly, for those who were living with someone else in 1991, we investigated characteristics of their primary coresident, which might be associated with ability to provide care at home for a cancer sufferer. Primary coresidents were selected using a hierarchical algorithm in which spouses, adult children, parents, siblings and other relatives were selected as the primary coresident in turn. In cases where there was more than one person in a particular category (for example, two adult children), we chose the oldest, and in the very few cases where two were of the same age we selected the woman, as being more likely to adopt a care-giving role. We distinguish between spouse primary coresidents and other primary coresidents and examine the distribution of each category by broad age group, whether or not they had a limiting long-standing illness and whether or not they had a full-time job. We also determine whether there was another adult in the household, who might be a potential support for both the person with cancer and the primary co-resident, and whether the household included a child under 18 years, who might present a competing care need.

Finally, we analysed variations in the proportions of decedents, 1991–1995, who died at home by whether they lived alone or with someone else in 1991; by health status of the coresident where there was one; by socioeconomic status; and by whether underlying cause of death was coded as cancer or not. The socioeconomic indicators drawn from the census were housing tenure (owner or renter) and whether electoral ward of residence fell into the top three or bottom two quartiles of Carstairs' index of deprivation (Carstairs and Morris, 1989). Information on date, place and cause of death (coded here using the ICD-9 classification with three digit codes) is available from linked death registration records. We calculated proportions of home deaths among LS members who died between the 1991 Census and the end of 1995 by sociodemographic characteristics, coresident's health and employment status and whether underlying cause of death was cancer. We then used logistic regression to analyse associations between presence, type (spouse or other) and health status of primary coresident with the proportion of decedents dying at home, including in the models gender, age group and socioeconomic indicators. This analysis was carried out for cancer deaths, noncancer deaths and all deaths combined in order to see whether effects varied by whether or not cancer was the cause of death.

## RESULTS

### Family and household circumstances of people with cancer

The 1981 sample analysed included 4238 people aged 50 years and over, who had been diagnosed with cancer in the previous decade; the equivalent 1991 sample of 6257 people was larger, reflecting the increase in the size of the older population and in the prevalence of cancer already referred to. Those living alone comprised 22.4 and 27.9%, respectively, of the 1981 and 1991 samples. As shown in Table 1

**Table 1:** Family/household type of people with a cancer diagnosis in the previous 10 years in 1981 and 1991

	**Census point and age group**							
	**1981 (%)**				**1991(%)**			
	**50–59**	**60–69**	**70–79**	**80+**	**50–59**	**60–69**	**70–79**	**80+**
*Men*								
Solitary	6.7	9.1	15.1	21.8	7.3	14.3	17.8	27.5
Couple	36.9	60.9	65.8	45.0	42.6	60.7	66.0	48.3
Couple+others	46.8	20.6	7.0	4.7	42.6	17.8	7.9	4.1
Lone parent	2.3	2.3	1.8	2.8	1.3	0.8	1.6	2.6
Complex	4.4	6.7	8.7	17.5	4.0	4.7	3.8	7.4
Communal est.^a^	<1	<1	1.5	8.1	<1	1.1	2.6	9.6
*All*^b^ (=100%)	344	608	654	211	399	859	1003	542
								
*Women*								
Solitary	9.3	27.6	41.3	45.2	12.9	26.9	46.7	55.1
Couple	43.7	47.4	33.3	10.2	44.8	49.2	34.1	11.6
Couple+others	35.6	9.3	4.9	1.4	30.2	10.6	4.2	0.4
Lone parent	4.7	5.4	5.8	9.1	6.7	6.4	4.2	3.9
Complex	4.2	10.0	11.0	22.4	4.1	6.3	7.4	11.7
Communal est.^a^	<1	<1	3.8	11.7	<1	0.3	3.2	17.1
*All*^b^ (=100%)	593	703	773	352	612	1011	1089	742

a Some results rounded in order to comply with confidentiality requirements.

b Including small numbers in categories not shown such as adult children living with parents.

, living arrangements of people with cancer varied considerably by age and gender. In both 1981 and 1991, over 80% of men with cancer aged 50–59 years lived with a spouse or a spouse and others, and although the proportion living alone or in complex types of family increased with age, even among those aged 80 years and over half lived with a spouse. Among women, the proportions living alone were much higher and the proportions living with a spouse much lower, as would be expected given gender differences in marital status distributions in older age groups. Among both men and women, the proportions living alone were higher, and proportions living with a relative other than a spouse lower, in 1991 than in 1981, especially in the oldest groups. In 1991, only 7% of men aged 80 years and over with a cancer registration lived in a complex household compared with 18% in 1981. A total of 55% of women aged 80 years and over who had cancer lived alone in 1991 and only 16% in a household including a relative other than, or in addition to, a spouse, compared with 45 and 33% respectively in 1981. These distributions and the differences between 1981 and 1991 are close to those noted in the whole population, and comparable data for the population without a cancer registration showed virtually no differences between those with and those without cancer (see appendix).

### Primary coresidents of people with cancer in 1991 (Table 2)

**Table 2:** Characteristics of primary coresidents of people with cancer, 1991

	**Age group and sex of person with cancer**									
	**Men (%)**					**Women (%)**				
	**50–69**	**70–79**	**80+**	**50+**		**50–69**	**70–79**	**80+**	**50+**	
**Characteristics of primary coresident (% with stated characteristic)**				**%**	***N***				**%**	***N***
	*Spouse primary coresident*									
Aged <50 years	11.7	<1	0	5.9	122	2.2	0	0	1.5	24
50–74 years	87.9	76.0	22.0	74.4	1537	94.3	41.9	4.6	75.4	1197
75+ years	0.5	23.8	78.1	19.7	407	3.5	58.1	95.5	23.1	366
Has long-term illness	20.6	33.5	44.6	28.6	591	27.4	41.2	48.9	32.3	512
Employed FT	17.6	1.5	<1	9.4	194	37.5	2.4	<4	26.2	416
No other adult in household	71.2	89.1	91.6	80.5	1663	74.1	88.3	95.5	79.0	1254
Child aged <18 years in household	8.4	1.6	1.1	4.9	101	5.4	<1	0	3.8	60
*N* (=100%)	1030	749	287	2066	2066	1079	420	88	1587	1587
										
	*Other primary coresident*									
Male	36.2	43.8	38.0	39.1	61	52.7	51.6	46.5	51.6	216
Female	63.8	56.3	62.0	60.9	95	47.3	48.4	53.5	48.4	210
Aged < 50 years	41.4	45.8	30.0	39.1	61	76.6	49.2	23.7	49.2	232
50–74 years	31.0	37.5	56.0	41.0	64	14.9	30.7	52.6	30.7	126
75+ years	27.6	16.7	14.0	19.9	31	8.5	20.2	23.7	20.2	68
Has long-term illness	27.6	41.7	18.0	28.9	45	14.9	22.6	29.0	22.6	89
Employed FT	20.7	33.3	38.0	30.1	47	52.1	41.1	36.8	41.1	191
No other adult in household	67.2	75.0	52.0	64.7	101	78.2	71.8	57.0	71.8	301
Child aged <18 years in household	17.2	10.4	12.0	13.5	21	16.5	5.7	8.8	5.7	48
*N* (=100%)	58	48	50	156	156	188	124	114	426	426

Note: Some results are rounded to comply with confidentiality regulations of the Longitudinal Study.

For the 4235 sample members who were living with someone else in 1991, we analysed characteristics of their primary coresidents. Most of this group (86%) lived with a spouse. The remaining 582 individuals had another primary coresident; 58% of these were adult children, 5% were parents, 12% siblings and 25% other relatives or unrelated persons. In most cases (78%), the LS member with cancer lived with one other person only and the small proportion in households including another adult decreased with age.

There was a strong association between the age group of the cancer sufferer and the age group of their spouses (*λ*^2^=76.79, *P*<0.0001). Thus, 78% of wives of men with cancer aged 80 years or more were themselves aged 75 years or more. Husbands of women aged 80 years and over with cancer were even more likely to themselves be aged at least 75 years. Related to this, spouse coresidents of the oldest LS members with cancer were the most likely to have a limiting long-term illness. The smaller group of non-spouse primary coresidents included a much lower proportion aged 75 years or over, not surprisingly given that many of these coresidents were children of the person with cancer. Even so, 23% of these other primary coresidents had a limiting long-standing illness. Overall, 1237 primary coresidents (26%) had a limiting long-term illness. However, this was no different from the expected number (1350, 28%) calculated using age- and sex-specific rates of limiting long-term illness.

### Living arrangements and death at home

Table 3

**Table 3:** Proportion of decedents who died at home in 5-year period following 1991 Census for cancer compared to other deaths by individual and household characteristics in 1991^a^

	**Cancer deaths**		**Other deaths**		**All deaths**	
**1991 characteristics**	**No. of deaths**	**% occurring at home**	**No. of deaths**	**% occurring at home**	**No. of deaths**	**% occurring at home**
Male	3400	30.5	8216	25.7	11616	27.1
Female	2922	24.6	8424	21.1	11346	22.0
50–59 years	837	36.1	1153	29.1	1990	32.0
60–69 years	1967	31.9	3279	29.9	5246	30.7
70–79 years	2345	26.1	6275	24.1	8620	24.6
80+ years	1173	18.5	5933	17.9	7106	18.0
Lives alone	1919	15.4	6311	20.8	8230	19.5
Lives with spouse with no long-term illness	2523	36.1	4974	25.5	7497	29.1
Lives with spouse with long-term illness	1227	30.3	2954	24.7	4181	26.3
Lives with other with no long-term illness	507	29.4	1801	24.2	2308	25.4
Lives with other with long-term illness	146	21.2	600	23.5	746	23.1
Primary coresident employed FT	762	35.6	1507	26.2	2269	29.4
Primary coresident not employed FT	3641	32.7	8822	24.7	12463	27.1
Other adult in household	935	37.0	2437	25.0	3372	28.4
No other adult in household	5387	26.2	3278	23.1	4689	23.9
Child aged <18 years in household	229	38.4	563	23.6	792	27.9
No child <18 years in household	6093	27.4	16077	23.4	22170	24.5
Owner occupier	3809	30.7	9529	24.7	13338	26.4
Tenant	2513	23.3	7111	21.6	9624	22.1
Carstairs quintile (1–3)	2824	29.9	7470	24.3	10294	25.8
Carstairs quintile (4–5)	3498	26.1	9170	22.6	12668	23.6
All	6322	27.8	16640	23.4	22962	24.6

a This analysis excludes those in communal establishments in 1991.

shows the proportion of home deaths among LS members who died between the 1991 Census and the end of 1995 by sociodemographic characteristics, coresident's health and employment status and whether the underlying cause of death was cancer. A total of 28% of those dying from cancer died at home, very similar to the 26% reported for cancer deaths among people of all ages in England in 1992 (Higginson *et al*, 1998). The proportion of home deaths was lower among women than men, decreased with age and was lower for those living alone than for those living with someone else.

Table 4

**Table 4:** Results from logistic regression model of proportion of decedents dying at home (in 5-year period following 1991 Census) from cancer or other causes by age, gender, type and health status of primary coresident; housing tenure and deprivation level of area of residence

	**Cancer deaths**		**Other deaths**		**All deaths**	
**Covariate (in 1991)**	**OR**	**95% CI**	**OR**	**95% CI**	**OR**	**95% CI**
60–69 years	0.90	0.75–1.07	1.07	0.92–1.24	0.97	0.87–1.09
70–79 years	0.75**	0.63–0.90	0.80**	0.69–0.92	0.75***	0.67–0.83
80+ years	0.56***	0.46–0.70	0.55***	0.47–0.64	0.52***	0.47–0.59
Female	0.92	0.81–1.03	0.85***	0.79–0.92	0.88***	0.83–0.94
Lives with spouse with no long-term illness	2.53***	2.15–2.97	0.97	0.88–1.07	1.28***	1.18–1.39
Lives with spouse with long-term illness	2.14***	1.79–2.56	1.05	0.94–1.17	1.25***	1.14–1.37
Lives with other with no long-term illness	2.13***	1.69–2.68	1.16*	1.02–1.32	1.32***	1.18–1.47
Lives with other with long-term illness	1.37	0.90–2.08	1.12	0.92–1.37	1.17	0.98–1.40
Tenant	0.86*	0.76–0.97	0.87***	0.81–0.94	0.86***	0.81–0.92
Carstairs quintile 4–5	0.89*	0.79–1.00	0.90**	0.84–0.97	0.90**	0.85–0.96

Reference categories: aged 50–59 years; male; lives alone; owner occupier; lives in area in top three quintiles of Carstairs score distribution.

* *P*<0.05;

** *P*<0.01;

*** *P*<0.001.

95% CI=95% confidence interval; OR=odds ratio.

presents results from regression analysis, showing odds of death at home by primary coresident's age and health characteristics and socioeconomic indicators (other variables shown in Table 3 were dropped as they proved nonsignificant). Among those dying of cancer, odds of a home death were highest (compared with the reference category of those living alone) for those living with a spouse who had no long-term illness (odds ratio (OR) 2.53, 95% confidence interval (CI) 2.15–2.97) and significantly raised for those living with a spouse who had a long-term illness (OR 2.14, CI 1.79–2.56) or with another adult with no long-term illness (OR 2.13, CI 1.69–2.68). However, for those dying from other causes, presence of a spouse with or without long-standing illness, or other primary coresident with a long-term illness had no significant effect, and having as a primary coresident someone other than a spouse with no long-term illness only a very marginal one (OR 1.16, CI 1.02–1.32, *P*<0.05).

Results from the model also showed that among both those dying from cancer and those dying from other causes, older age was associated with reduced odds of a home death (even though living arrangement was included in the model) as were indicators of socioeconomic disadvantage. Those dying aged 70–79 or 80 years and over were much less likely to die at home than people in their 50s or 60s; compared with the reference category of people aged 50–59 years at death, people dying of cancer at ages 80 years and over were only just over half as likely to die at home (OR 0.56, CI 0.46–0.70). Tenants and people living in wards in the two most deprived quintiles of the Carstairs' distribution also had lower odds of a home death than owner occupiers and those living in more advantaged areas.

It is possible that these results were influenced by variations between groups in the distribution of types of cancer. However, when we performed the same analyses for grouped categories of people dying from cancers with very poor prognosis and those dying from other cancers, we found no differences between the two.

## DISCUSSION

These results from a large nationally representative study show that the living arrangements of older people with cancer, and trends in these, are very similar to those for the rest of the population. By 1991, over half of female cancer sufferers aged 80 years and over, and a quarter of equivalent men, lived alone. Although these results indicate a lack of family support available within the households of many older cancer sufferers, our data do not allow us to identify familial support from non-coresidents, so these results should not be interpreted as indicating an overall lack of family support for older people with cancer. We cannot, for example, identify cancer sufferers who were supported by relatives taking it in turns to stay with them overnight, rather than moving in. Of those cancer sufferers who lived with someone else, their primary coresident was in most cases a spouse, with much smaller proportions living with a child, a sibling or another person. In all, 30% of spouse coresidents and 23% of other coresidents had a limiting long-term illness and 21% of the former and 17% of the latter were themselves aged 75 years or over. Presence, type and health status of primary coresident were associated with differentials in the proportions of people dying of cancer who died at home, but not to the same extent with home death among those dying of other causes. This difference may be because cancer deaths are more often anticipated than deaths from other causes so death at home may be planned, given appropriate support. Older age and lower socioeconomic status, both individual and area, was associated with reduced chance of a home death for both cancer deaths and deaths from other causes.

Data from the 2001 Census have not yet been added into the ONS LS, but we know from other sources that among people aged less than 75 years, the proportions living with a spouse increased reflecting a slight narrowing of sex differentials in mortality and consequent delay in widowhood (Tomassini *et al*, 2004). However, in the population aged 75 years and over – which is rapidly growing – the trend towards residential independence continued throughout the 1990s, albeit at a slightly slower rate. As half of all cancer deaths occur among people aged 75 years and over (National Statistics, 2001), this implies an increasing challenge to the delivery of home-based care and more pressure on hospital beds and other locations, such as hospices, for terminal care. Indeed our results may partly explain why it is that although most cancer patients express a wish for a home death, until recently the proportion of home deaths has been decreasing (Higginson *et al*, 1998).

The UK health secretary recently voiced a commitment to ensuring that palliative care is available to all who want it and the National Health Service has launched a pilot programme to test various at-home models for terminally ill people (Burke, 2004). It is important that such initiatives take into account the changing living arrangements of older people, changes which also have implications for the quality of life of cancer sufferers.

## Appendix

See Table A1

**Table A1:** Family/household type (%) of people with no cancer diagnosis in the previous 10 years in 1981 and 1991

	**Census point and age group**							
	**1981 (%)**				**1991 (%)**			
	**50–59**	**60–69**	**70–79**	**80+**	**50–59**	**60–69**	**70–79**	**80+**
*Men*								
Solitary	6.9	10.5	17.6	27.8	9	13.3	19	30.1
Couple	31.5	56.8	59.9	40.5	35.9	58.1	62	46.1
Couple+others	48.8	22	9.4	5.3	44.2	19.5	9	4.4
Lone parent	2.5	1.7	2	3.9	2.1	1.6	1.7	2.4
Complex	7.4	7.3	8.7	15	6.1	5.8	5.7	7.5
Communal est.^a^	1.2	1.1	2.5	7.6	0.6	0.9	2.4	9.2
*All*^b^ (=100%)	29 670	24 087	14 070	3623	28 442	24 465	14 961	5128
								
*Women*								
Solitary	9.2	24.6	44	47.2	11.1	24.6	45.1	51.9
Couple	37.3	47.6	31.1	9.9	42	51.1	34.9	12.1
Couple+others	38	12.3	3.6	1.4	33	11.4	3.6	1
Lone parent	6.7	5.1	5.4	7.2	6.6	4.9	4.7	5.2
Complex	6.7	9.1	12.9	20.9	5.8	6.6	8.1	11.5
Communal est.^a^	0.5	0.9	2.9	13.3	0.4	0.8	3.2	18.1
*All*^b^ (=100%)	30 425	28 131	21 463	9564	27 705	27 086	21 778	12 577

a Some results rounded in order to comply with confidentiality requirements.

b Including small numbers in categories not shown such as adult children living with parents
